# Journalistic

**Published:** 1890-02

**Authors:** 


### Journalistic Notes.

THE ST. LOUIS POLYCLINIC has changed its name to the *Courier of Medicine.*

---

THE DIXIE DOCTOR is the name of a new journal that is published at Atlanta, Ga. It is edited by T. H. Huzza, M. D.

